# Age-Related Cognitive Impairment as a Sign of Geriatric Neurocardiovascular Interactions: May Polyphenols Play a Protective Role?

**DOI:** 10.1155/2015/721514

**Published:** 2015-06-09

**Authors:** Fedor Jagla, Olga Pechanova

**Affiliations:** Institute of Normal and Pathological Physiology, Slovak Academy of Sciences, Sienkiewiczova 1, 813 71 Bratislava, Slovakia

## Abstract

It is known that endothelial dysfunction plays an important role in the development and progression of cardiovascular diseases implicated also in cognitive decline. Experimental studies pointed to the fact that the modification of NO levels via NOS activity may affect the blood pressure level as well as several higher nervous functions—for example, learning and memory. There are emerging evidences from *in vitro* and animal studies suggesting that polyphenols may potentially have a protective effect on the development of neurodegenerative diseases and may improve cognitive function as well as positively affecting the blood pressure regulatory mechanisms. This review accentuates the need for precisely defined clinically controlled studies as well as for use of adequate experimental procedures discriminating between the human higher brain functions and the only overall activation of the brain cortex. The physiological neurocardiovascular interactions are implicated in the increased healthy life span as well.

## 1. Introduction

The European health report of WHO from 2012 points to the fact that the group of people aged 65 or more years constituted 15% of the total by the end of 2010 and is projected to comprise more than 25% by 2050. Consequently, related health care costs will rise significantly. Therefore, it is important to search for possibilities to maintain health and cognitive health with age as well. It is referred that 25–30% of people aged 85 or older have some degree of age-associated cognitive decline which may significantly influence their instrumental activities of daily living [1]. Age-associated cognitive decline differs in extent among the individuals. The cognitive decline is one of the most feared aspects of becoming old. Cognitive functions are under the influence of many factors and their mechanisms are poorly understood. The aging and cardiovascular diseases are either directly or indirectly responsible for cognitive decline. The age related cognitive decline which is in relation to cerebrovascular dysfunction in senescence becomes a very serious problem of geriatric medicine and care. The pathological studies have pointed to the fact that up to the 34% of dementing illnesses show postmortem confirmed significant vascular pathology [2]. The identification of factors helping people maintain their cognitive health becomes the very important current public health challenge.

## 2. NO and Basic Neurocardiovascular Interactions

It is now known that a lot from all biochemical factors modulating the blood flow regulate also the neuronal functions including the freely diffusing nitric oxide (NO) molecule [3]. Patterning and branching of blood vessels and nerves are strongly molecularly linked [4]. Diffusible messengers can directly impact neural activity which increases with increased blood volume. That is, microvascular endothelial cells participate in signal processing in the brain by generating tonic and phasic NO signals; the endothelial NO production leads to a net depolarization in proximal nerve fibers [5]. It is a very important finding when assuming the fact that the brain functions are made possible by interconnected structure of neurons, glia, and microvessels. To do it the brain utilizes over 100 billion cells and 600 km of micro vessels [6]. The neurons, glia, and vascular cells meet in the so-called neurovascular unit which is the basic structural and functional brain unit for information processing, integration as well as energy supply. All brain networks consist of three compartments, neuronal, glial, and capillary, forming the brain complex cellular networks. This complex cellular network is a base for the brain functional hyperaemia which is known as the increased blood flow and volume within the activated brain region [7]. Moreover, the endogenous NO production in the thalamus, which is very important in the gating mechanisms, varies with the behavioural state [8]. It points to the important role of NO within the regulatory mechanisms for interacting components of neuronal networks coherent firing of which creates a basis for the human higher brain functions, that is, for cognitive, strategic, and affective neuronal networks mainly. Their proper regulatory mechanisms are very important concerning the healthy aging.

Healthy aging and physical well-being depend in a great extent on properly functioning vascular and nervous system. There is a bulk of literature describing the beneficiary effects of the polyphenols upon the cardiovascular system. Their ability to improve endothelial function by increasing production of the signalling nitric oxide molecule leading to relaxation of the endothelial smooth muscle resulting in a greater control of blood pressure was documented repeatedly. Increased NO level inhibits the enzymes like cyclo-oxygenase (COX-2), reactive C protein, and the atheromatous plaque adhesion molecules known to be involved in inflammation [9]. Endothelial dysfunction plays a role in the development and progression of cardiovascular diseases implicated in cognitive decline and is also an important mechanism to be considered in development of neurodegenerative disorders [10]. In the review article, Vauzour et al. [11] pointed to the suggested mechanism for the action of polyphenols on vascular function which involves their ability to modulate the levels of and activity of nitric oxide synthase (eNOS) and therefore nitric oxide (NO) bioavailability to the endothelium. This regulation of vascular nitric oxide is thought to involve the ability of polyphenols to interact with kinase signaling pathways such as the PI3-kinase/Akt pathway and intracellular Ca^2+^ on eNOS phosphorylation and subsequent NO production. They also pointed out that polyphenols were shown to act to prevent age-related vascular injury. Recently, Valls-Pedret et al. [12] have shown that increased consumption of polyphenols in the group of 447 elderly subjects at high cardiovascular risk was associated also with better cognitive performance. The effect of polyphenols on cognitive functions is as a hot topic now the subject of the randomized, placebo controlled, double-blinded clinical trials [13].

On the other hand, the essential hypotension has been shown to cause prolonged execution times in the attention tasks; moderately decreased accuracy was found in the tests assessing sustained attention and working memory and hypotensive subjects showed smaller elevations in BP during the execution of the cognitive tasks [14]. Moreover, relentless brain hypoperfusion may be responsible for protein synthesis abnormalities that later result in neurodegenerative lesions and thus induce progressive cognitive impairment [15]. Unfortunately, the cognitive decline due to the hypotension is mostly ignored. The age-related cognitive impairment as seen at subtly diminished some neuropsychological functions [16, 17] may be taken as an indicator of the level of the geriatric neurocardiovascular interactions.

## 3. Polyphenols and Brain Regulatory Mechanisms

The role of diet and other lifestyle factors in successful aging belong to the current topics of scientific interest [18]. Several findings suggest that both the physiological and psychological diet components might help to delay the onset and/or slow the progression of age-associated cognitive decline [19]. Oxidative damage, among others, is implicated in aging and age-associated cognitive decline. Dietary antioxidants found in fruits and vegetables may help protect against oxidative damage and several studies support a link between the antioxidant status and cognitive functions in senescence [20]. The polyphenols are proposed as the mostly rich natural substances with antioxidant potential in our diet [21].

The neurological benefits of polyphenols, especially of resveratrol, a potential antioxidant from red wine polyphenols, were documented experimentally in cerebral ischemia, brain oedema, Parkinson disease, amyotrophic lateral sclerosis, brain tumours, pain, cognitive impairments, aging, and several other CNS disorders [22]. According to Hollman [23], there is no doubt that polyphenols are excellent antioxidants* in vitro*, but systemic antioxidant effects* in vivo* are hard to prove. The preclinical studies have shown that polyphenols display neuroprotective effects, enhance neuronal functions, stimulate brain flow, induce neurogenesis and prevent age-related damage through their antioxidant and anti-inflammatory activities. Kesse-Guyot et al. [24] have pointed out that high intake of specific polyphenols may help to preserve verbal memory which is a salient vulnerable domain in pathological brain aging. As Vauzour et al. have stressed [11] the polyphenols can cross the blood brain barrier but the question of their dose remains to be answered. The effects of polyphenols upon the brain functions are associated with nitric oxide signalling. The effects of polyphenols on cognition and against neurodegenerative processes appear to be mediated via their interactions with neuronal and glial signaling pathways that affect gene expression and interfere with the cell death mechanisms. One NO signalling pathway affects the brain derived neurotrophic factor (BDNF), secretory protein, neurophilin, which regulates neuronal surviving and differentiation. It means that BDNF affects the axonal and dendritic growth and via such a way the synapses and components of release of the neurotransmitters [25, 26] NO act in a positive feedback loop with BDNF to regulate neural progenitor cell proliferation and differentiation in the mammalian brain. BDNF stimulates nNOS and NO, produced by the proliferating cells, and signals to begin differentiating into neural cell phenotypes [27]. The regulation of neuronal growth and synaptic metabolism is essential for cognitive functioning.

There exist a small number of clinical trials about the therapeutic benefits of the natural substances. It is also known that the diet and the other important factors of lifestyle are not so exactly controlled as in animal studies. But Letenneur and coworkers [28] published the results of PAQUID study (Personnes Agées Quid) on prospectively examined flavonoid intake in relation to cognitive functions and decline. At the start of study, 1640 subjects aged 65 or older free from dementia were adjusted for age, sex, and educational level. During the following 10 years' period, they were four times reexamined at home by psychologist by means of Mini-Mental State Examination, Benton's Visual Retention Test and “Isaacs” set test and a comprehensive dietary survey was performed. Subjects within the two highest quartiles of flavonoid intake had significantly better cognitive functions as well as evolution of performance over time.

**Figure 1:**
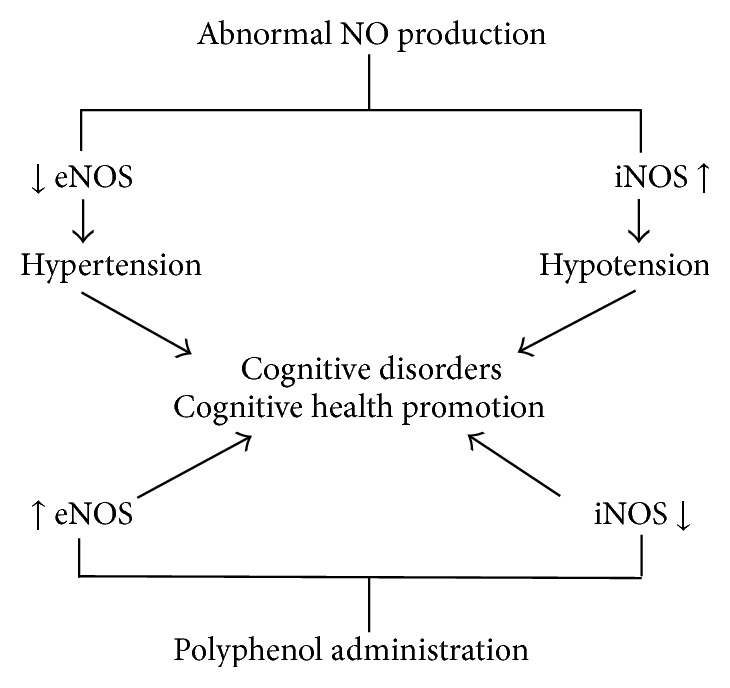
Changes of eNOS and iNOS activity and their effects upon human cognitive function.

It is now known that dietary polyphenols are extensively metabolized* in vivo* and the chemical, biophysical, and biological properties of their metabolites are quite different from those of parent molecule. The systemic effects of polyphenols are associated with nitric oxide signalling. Actually, NO plays a dual role in tissues and cells. It belongs to essential physiological signalling molecule mediating various cell functions but when present in excess it induces cytotoxic and mutagenic effects [29], as for neurons also [30]. While the normal production of NO is associated with normal function of eNOS its overproduction (toxic) is associated with increase in inducible NOS (iNOS). Results of many studies demonstrated clearly that inhibition of endothelial nitric oxide synthase (eNOS) reduces blood flow and may contribute to cognitive dysfunction [31]. Cai et al. [32] stated that the downregulation of iNOS and upregulation of eNOS may protect from cognitive impairment.  Figure 1 shows how the changes of eNOS and iNOS activity can affect human cognitive functions. Animal model studies pointed to the fact that the modulation of NO levels via NOS activity, for example, by administration of polyphenols, may affect aging and dementia [33], memory deficits and long-term memory [34], spatial working memory [35], and performance in cognitive tasks relating to learning and memory as well [36].

Moreover, the role of NO and NOS was described in psychiatric disorders also. Several studies have shown a prominent role of NO in the pathogenesis of major depression. Nitric oxide modulates norepinephrine, serotonin, dopamine, and glutamate, the major neurotransmitters involved in the neurobiology of major depression [37]. The role of NO and NOS was described in schizophrenia as well [38, 39] and an increased level of NO was characterized as a link between cognitive impairment in Alzheimer disease patients [40]. In the human studies, the question of genetic polymorphisms of relation between NO and psychiatric disorders was neglected. Quite recently, it was found that the variability in nNOS gene is associated with schizophrenia [41]. A functional promoter polymorphism in neuronal NOS (nNOS) was proposed to be associated with personality traits related to impulsivity [42]. It may open new field for study disorders with difficulty to control impulses as primary feature (e.g., kleptomania, pyromania, and others) and disorders with difficulty to control impulses that is not their primary feature as for example, attention-deficit/hyperactivity disorder, or manic state of bipolar disorder [43].

Several of our own studies have shown [44–47] that administration of polyphenols may evoke following effects in the animal brain: (1) antioxidant activity, mainly inhibition of the NADPH oxidase and subsequent reactive oxygen species generation; (2) an activator effect on endothelial and inhibitory action upon both neuronal (nNOS) and inducible nitric oxide synthase activity; (3) downregulation of the proinflammatory transcription factors such as NF-*κ*B; and (4) modulation of signalling pathways such as mitogen-activated protein kinase cascade and cAMP response element-binding protein leading to the improvement of memory and cognitive performance. Nevertheless, the question still remains whether the polyphenols may have also an influential general beneficial effect in relation to the behavioural and brain functions of senior persons.

## 4. Polyphenols and Cognitive Functions

Weichselbaum and Buttriss [48] pointed to the fact that there is emerging evidence from* in vitro* and a few animal studies suggesting that polyphenols may potentially have a protective effect on the development of neurodegenerative diseases and may improve cognitive function in patients when such diseases are established but the controlled human studies are needed. At the same time Macready et al. [49] reviewed 15 human randomized controlled trial studies on the effect of flavonoids upon the cognitive functions. As the authors pointed out, the findings from this selection of human cognition studies using flavonoid treatments are suggestive of a positive association between flavonoid consumption and cognitive function. The reviewed studies employed a total of 55 different cognitive tests covering a broad range of cognitive domains and most studies incorporated at least one measure of executive function/working memory, with nine reporting significant improvements in performance as a function of flavonoid supplementation compared to a control group. This publication clearly draws attention to the fact that to characterize the influence of biologically active substance upon the cognitive functions requires exact definition of the measured executive function, type of memory, motor function with or without a cognitive component, and also type of measured IQ. Consequently, the authors stressed the fact that a great deal of work still needs to be carried out to identify tasks that are sensitive to flavonoid-related cognitive changes in healthy human populations. Moreover, it seems that there may exist differences in cognitive decline related to the consumption of nutrients available in fruits and vegetables [50]. Our own results suggested also the need to use adequate experimental procedures for discriminating among such higher brain functions as perception, memory, and attention as opposed to the only overall activation of the brain cortex [51, 52].

## 5. Polyphenols and Visual-Oculomotor Integration

In our preliminary study concerning the effect of single polyphenol substance administration in normotensive young healthy persons, we hypothesized that it may positively influence some human higher brain functions which are usually mostly affected in age-cognitive decline and/or disorders of the central nervous system [52]. Because the interaction between subjects, between subject and animal, and also between subject and environment is subserved by sensory-motor integration and the most tight and subtle one is the visual-oculomotor integration we decided to analyse the accuracy of saccadic eye movements. The saccades are the rapid jumps of eyes by means of which the subject is scanning the visual environment. In our experimental examinations, we recorded the saccadic eye movements by means of the electrooculography. At the beginning of examinations, the saccades were elicited by switching on the 0.3° circular visual targets in the visual field (the visual-guided saccade task—VGS). The subject has to fixate as rapid and accurate as possible the visual targets which appeared suddenly in the visual field. The subjects are not aware of the control mechanisms for execution of these reflexive movements. But one can split the visual-oculomotor integration by using the memory-guided saccades paradigm. Immediately after the end of the VGS task the memory-guided saccade task (MGS) was introduced [53]. Subjects have to fixate a central visual target and to continue its fixation while another visual stimulus was briefly flashed into the periphery of the visual field. He/she has to remember the location of the peripheral visual stimulus. After the central fixation target was switched off subject had to make a saccade to the remembered peripheral target location. Following the registration of VGS and MGS tasks in one group of volunteers the polyphenolic substance extracted from red wine (Provinols, 4 mg/kg of body weight) was administered; in the second group the placebo was used and in the third group nothing was administered. The whole procedure was repeated two and three hours later (the composition of Provinols was as follows: proanthocyanidins 480, total anthocyanidins 61, free anthocyanidins 19, catechin 38, hydroxycinnamic acid 18, flavonols 14, and polymeric tanins 37 mg/g of Provinols).

In average, the 95% of VGS are accurate contrarily to 50–60% of MGS. Two hours after the administration of Provinols, the accuracy of MGS was substantially and significantly increased but only slightly of VGS. The results confirmed the significantly increased number of more accurate memory guided saccades after the Provinols administration, which suggested a better performance in the MGS test. Nevertheless, the question remains what actually the administration of the polyphenolic substance affected: spatial memory, working memory, encoding of visual stimuli, execution of MGS, visual perception, or attention.

Another interesting finding concerns the relation of horizontal saccadic eye movements to the functional brain asymmetry. The VGS directed to the motor dominant hemisphere are more accurate—they are followed by less number of corrective saccades. Moreover, when the reflexive saccade toward the visual target is inaccurate and is followed by a corrective saccade the corrective ones are of significantly shorter latency when directed towards the motor dominant hemisphere. The shortest latency is recorded when the correction is directed from the periphery of the visual field towards its centre [54]. In this case, the functional brain asymmetry helps the person to catch and process the visual stimulus as early and as accurate as possible. This relation is not present after the administration of Provinols. Similar finding we had seen in patients suffering from depression and panic disorder [55, 56]. Administration of Provinols increased the overall activation level of the brain cortex [57]. The question is open about what is done with NO signalling when the level of activation and/or excitation is enhanced by intensive focusing of the attention on the subjective problems or by administration of some biologically active substance as, for example, Provinols?

In a subsequent study, the whole experimental design was repeatedly used and, in addition to the electrooculography, the electroencephalography and the blood pressure changes were recorded [50]. In general, the accuracy of VGS and MGS saccades was the same as in previous study. No differences in EEG evoked potentials time-locked to saccadic eye movement onsets among the three groups of subjects were found. There were no differences in preparation and execution of saccades as well as in the time of first encoding the new visual information within the primary visual cortex. As expected, the single administration of polyphenolic substance did not affect the blood pressure. As for the EEG power spectral densities, during the preparatory and execution periods for saccades guided by memory information the significant decrease within the slow EEG bands was registered, alpha power mainly. It means the after the Provinols administration the heightened cortical activation appeared in areas playing the role in attention spatial orienting and memorizing as well.

Up to now, it is not possible to separate attention and memory components in our results but they clearly demonstrate that the polyphenolic extract from red wine Provinols positively affects some cognitive/integrative higher brain functions and probably the attention and activation status of the human brain as well. Taking into the consideration also the above mentioned results of PAQUID study by Letenneur et al. [28], the preventive as well as activation effects of polyphenols upon human cognitive functions are quite promisingly suggested at least in young healthy persons. In order to differentiate the effects of polyphenols upon the cognitive, affective and integrative human higher brain functions the more precise experimental set up should be introduced for the future studies. Because of the changes in metabolism rate in elderly subjects the question of the above mentioned effects of administration of polyphenolic substances upon the MGS have to be experimentally proved also.

## 6. NO, Human Aging Problems, and Life Span

Because the NO plays a significant role in vascularization and neurogenesis, it is quite obvious that some authors point to its possible effect on life span [58]. Cheng et al. [59] discovered that NO acts in a positive feedback loop with brain derived neurotrophic factor (BDNF) to regulate neural progenitor cell proliferation and differentiation in the mammalian brain. BDNF stimulates nNOS and NO produced by the proliferating cells signals to start differentiation into neural cell phenotypes. Ungvari et al. [60] summarized their review article pointing that increased bioavailability of NO, decreased vascular reactive oxygen species generation, activation the antioxidant response element pathway, induction of reactive oxygen species detoxification systems exert anti-inflammatory effects and, thereby, suppressed initiation and/or progression of vascular disease that accompany aging. The study of Montesanto et al. [61] has shown that genetic variability of NOS genes has an effect on common age related phenotypes and longevity in humans as well as previously reported for model organisms.

Physiological aging is accompanied by several mental changes which are more or less “normal” symptoms of aging as well as of old age. The dynamics of psychic activities decreases overall and central fatigue increases. The increased extracellular glutamate concentration was described in neurodegenerative and inflammatory brain diseases [62–64] and the potential role of glutamate transport in mental fatigue was hypothesized [65]. Hu et al. [66] found a dose-dependent inhibition of astrocyte glutamate uptake by a mechanism in which NO plays a role.

The human motor manifestations during aging are decreased also. It was reported that NO plays an important role in the control of behaviour [67] and locomotion as well [68–70]. The more recent human studies indicate that exercise training for a period ranging from days to several weeks enhances basal release of nitric oxide from the aorta, active and inactive muscle, and coronary arteries. This may contribute to the reduction in resting blood pressure that can be observed after as little as 4 weeks of training [71, 72]. Increased NO production with a wide range of intensities and duration of physical trainings that progresses to structural and other sustained adaptations were documented, [73, 74]. The positive effect of physical exercises upon the aging brain in clinical as well as nonpathological populations was also repeatedly documented [75] In spite of the fact that physical exercise promotes brain and cognitive vitality into older adulthood the more intervention research is needed as Kramer et al. [76] pointed out taking into the consideration also their previous meta-analysis of the relevant literature from 1966 to 2001.

Disorders of memory functions during aging steadily and gradually increase. General principles on how NO and memory are related were recently described by Susswein et al. [77]. However, the authors stressed the fact that the role of NO in memory formation is extremely variable. It seems to be strongly dependent not only on the particular animal investigated but also on the specific behaviour and training paradigm examined.

In the course of aging, the expansive and confrontational symptoms are losing and the depressive and anxiety reactions are coming to the fore. Of course, there are more “normal” symptoms of aging but in the majority of them the NO signalling may play a role. The NO and aging are closely related and it seems that NO system in each brain region may influence chemical and structural changes in the CNS during aging [78]. Moreover, it was pointed out that the mechanisms by which NOS enzymes promote vascular dysfunction in aging are specific for each enzyme isoform [79]. The question of the specific/general mechanisms of the effects of polyphenols in relation to healthy aging is still open to be solved.

Normal aging is a very complex process under the influence of many factors that vary from individual to individual. The main problem is how to change lifestyle and habits of older population. Further clinically controlled studies and adequate experimental procedures are needed. It is now clear that the multi-/transdisciplinary approach only may reveal the causal mechanisms underlying the neurocardiovascular interactions serving as a prerequisite for physiological aging, cognitive health promotion and a longer healthy life span. Such a demand is very important because of the need for controlled clinical trials to determine the effect of reducing vascular risk factors upon decreasing a risk for cognitive decline, improving cognitive function and lengthening the healthy life span [80].

## 7. Conclusion

For many people, aging is associated with little cognitive decline, for some memory declines significantly with age and for others aging is associated with severe cognitive deficits. Moreover, not all cognitive functions are affected equally by age. Several health factors and behaviours may be protective in maintaining cognitive function, nutrition being one of them. Our own data have shown that even the single administration of polyphenols may significantly increase the level of the overall activation of the brain cortex and performance in tests of cognitive functions; the effect is time limited. Literary data support the opinion that the chronic consumption of polyphenols rich diet can promote the “healthy” aging, that is, to delay the cognitive decline and to prolong the healthy life span and protect the good execution in daily activities of seniors. We have stressed the importance of further controlled clinical trials with very carefully defined cognitive tasks to be administered as also the use of the registration of appropriate sensorimotor function as a valid indicator of the effect of polyphenol administration and of the level of neurocardiovascular interaction of the aged person.
